# Glioblastoma Tumor Microenvironment and Purinergic Signaling: Implications for Novel Therapies

**DOI:** 10.3390/ph18030385

**Published:** 2025-03-08

**Authors:** Martina Bedeschi, Elena Cavassi, Antonino Romeo, Anna Tesei

**Affiliations:** 1 Biosciences Laboratory, IRCCS Istituto Romagnolo per lo Studio dei Tumori (IRST) “Dino Amadori”, 47014 Meldola, Italy; martina.bedeschi@irst.emr.it (M.B.); elena.cavassi@irst.emr.it (E.C.); 2 Radiation Oncology Unit, IRCCS Istituto Romagnolo per lo Studio dei Tumori (IRST) “Dino Amadori”, 47014 Meldola, Italy; antonino.romeo@irst.emr.it

**Keywords:** purinergic receptors, glioblastoma, tumor microenvironment, P2X7R, P2X4R, purinergic antagonists

## Abstract

Glial-origin brain tumors, particularly glioblastomas (GBMs), are known for their devastating prognosis and are characterized by rapid progression and fatal outcomes. Despite advances in surgical resection, complete removal of the tumor remains unattainable, with residual cells driving recurrence that is resistant to conventional therapies. The GBM tumor microenviroment (TME) significantly impacts tumor progression and treatment response. In this review, we explore the emerging role of purinergic signaling, especially the P2X7 receptor (P2X7R). Due to its unique characteristics, it plays a key role in tumor progression and offers a potential therapeutic strategy for GBM through TME modulation. We discuss also the emerging role of the P2X4 receptor (P2X4R) as a promising therapeutic target. Overall, targeting purinergic signaling offers a potential approach to overcoming current GBM treatment limitations.

## 1. Introduction

Glioblastoma (GBM) is the most frequent primary malignant brain tumor in adults, representing approximately 57% of all gliomas and 48% of all primary malignant central nervous system (CNS) tumors [1]. GBM is characterized by a median survival of less than 15 months. Its incidence increases with age, peaking between 75 and 84 years, with an age-adjusted incidence rate of 3.22 per 100,000 population [2].

The Stupp protocol, the current gold standard for GBM treatment, involves maximal safe surgical resection followed by radiotherapy and temozolomide (TMZ) chemotherapy [3]. However, it remains associated with substantial side effects and limited effectiveness in achieving long-term survival. The critical need for therapeutic advancements in GBM is underscored by the discouraging survival rates of 6.6% at 5 years post-diagnosis [4].

Efforts elucidating the genetic makeup of GBM [5,6] have thus far not resulted in the development of more effective treatments. New therapeutic approaches for gliomas, such as kinase inhibitors, alkylating agents, proteasome inhibitors, and transcription factor inhibitors, have failed to enhance overall survival over the past 20 years. Even advances in immunotherapy have yielded disappointing results. Recent clinical trials using PD-1 inhibitors in both recurrent and newly diagnosed GBM have also demonstrated no improvement in patient survival [7].

Indeed, GBM presents several challenges to effective treatment, including its aggressive infiltration into healthy tissue and the presence of the blood–brain barrier (BBB), which hinders both the delivery of therapeutic agents and the immune response [8,9]. Moreover, the immune system of the CNS, once thought to be isolated due to the BBB, lack of classic lymphatic drainage, and limited foreign tissue rejection, is now understood to be functional yet distinct, with a unique morphological architecture that shapes its immune responses [9].

In this intricate context, GBM is characterized by pronounced local and systemic immunosuppression, driven by the formation of a profoundly immunosuppressive tumor microenvironment (TME) and a cold immune phenotype. This distinctive TME not only suppresses endogenous antitumor immune responses but also hampers the efficacy of immunotherapy, rendering it unique in its composition and function [10,11].

Within the GBM TME, the role of purinergic signaling remains relatively understudied. A very recent work reported that in vitro and in vivo GBM models exhibited comparable trends, showing increased mitochondrial activity in tumor-associated macrophages and microglia (TAMs) alongside extracellular ATP (eATP) production in the presence of GBM cells. Tumor-secreted factors, such as granulocyte macrophage colony-stimulating factor (GM-CSF), were found to drive the rise in eATP production by TAMs. Furthermore, the elevated eATP levels within the GBM microenvironment facilitated glioma growth and invasion through the activation of the P2X purinoceptor 7 (P2X7R) on glioma cells [12].

This review aims to provide an overview of the current understanding of purinergic signaling in the GBM TME, with a special focus on the expression and function of P2X7R. Additionally, we will explore the potential of targeting GBM TME through the effectors of purinergic signaling as a novel therapeutic strategy for GBM.

## 2. Key Features of the Glioblastoma Tumor Microenvironment

**Figure 1:**
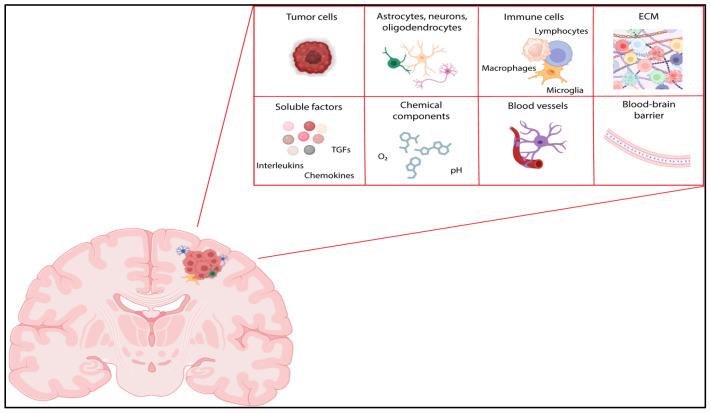
Glioblastoma tumor microenvironment. Glioblastoma (GBM) tumor microenvironment (TME) is composed of a diverse array of cell types, including glioblastoma cells, macrophages, monocytes, microglia (TAMs), tumor-associated neutrophils (TANs), astrocytes, endothelial cells, neurons, and the extracellular matrix (ECM). All the GBM TME components are closely interconnected, interacting through various mediators and chemoattractive processes.

The GBM TME is a highly heterogeneous and complex system, comprising not only cancer cells but also various resident brain and immune cells, as well as transiting cells, such as marrow-derived immune cells (Figure 1) [13]. The GBM TME is a dynamic environment that is shaped by changes in cellular composition, cell-to-cell interactions, metabolic by-products, and other chemical factors, including pH and oxygen levels [14]. Emerging evidence indicates that GBM cells reprogram the TME and exploit its components to promote rapid proliferation, invasion, migration, and survival, thereby contributing to treatment resistance [13]. In recent years, it has become a focal point of intense research, driven by the urgent need to develop targeted therapies that specifically address the TME as an alternative target to curb the relentless growth of GBM. Towards this direction, the recent work by White and colleagues [15] introduces a novel classification system for GBM based on the TME. Using a refined microenvironment cell population (MCP) counter method, the researchers identified three distinct TME subtypes: TME^High^, TME^Med^, and TME^Low^. TME^High^ GBMs, comprising 30% of the samples, are characterized by high immune cell infiltration and the expression of immune checkpoint proteins, suggesting a highly immunosuppressive environment. TME^Med^ GBMs, representing 46% of the samples, show heterogeneous immune populations and high endothelial cell gene expression. TME^Low^ GBMs, making up 24% of the samples, are described as “immune-desert” with low immune and endothelial cell abundance. The study strongly suggests that TME subtyping could guide the selection of patients for specific immunotherapy regimens in GBM. In particular, TME^High^ patients showed improved overall survival when treated with neoadjuvant anti-PD-1 therapy, indicating that TME subtypes can predict response to immunotherapy.

The following paragraphs will provide a synthetic description of both the non-immune and immune cell populations that contribute to the immunosuppressive and complex TME in GBM, specifically impacting the effectiveness of immunotherapies.

### 2.1. Immunocomponent Cells

Microglia. Glioma-associated microglia and macrophages (GAMs) represent one of the most abundant cell populations in the GBM TME, constituting 30–50% of the immune cells within the tumor mass [16]. The infiltrating GAMs play a key role in promoting tumor progression, either by enhancing cancer cell invasion or by inducing T-cell exhaustion, which contributes to the establishment of an immunosuppressive microenvironment that drives therapeutic resistance [17]. While several chemokine–receptor interactions, such as CCL2–CCR2, OPN–αvβ5 integrin, LOX–β1 integrin, and SLIT2–ROBO1/2, have been identified, the precise mechanisms regulating GAMs infiltration and survival remain poorly understood [18,19,20,21]. GAMs include diverse subpopulations, such as bone marrow-derived macrophages and brain-resident microglia. Recent studies aimed at distinguishing between microglia and recruited macrophages have sparked considerable debate about their distribution and functional roles within the tumor tissue. This complexity is further compounded by the fact that findings often vary depending on the methods used to differentiate these two cell types [22]. A significant challenge remains the limited tools available to distinguish microglia from macrophages, as they share similar surface markers and morphology.

Tumor-Infiltrating Lymphocytes (TILs). TILs, including CD4+ and CD8+ T-cells, are present in the TME but often exhibit a dysfunctional, exhausted phenotype due to the suppressive actions of TGF-β, IL-10, and CCL2. Glioma cells express molecules, like FAS ligand, PD-L1, and PD-L2, which suppress TIL function [23].

Natural Killer (NK). NK cells are a minor component of the GBM TME and are often non-functional due to interactions with TAMs, myeloid-derived suppressor cells (MDSCs), T-regs, and inhibitory ligands, like HLA-G and TGF-β expressed by GBM cells. Furthermore, Lee et al. demonstrated that NK cells play an important role in regulating GBM invasiveness [24].

Neutrophils. Regarding neutrophils, Maas et al. showed that in IDH-wildtype gliomas and brain metastases, a high number of neutrophils was found, and they displayed a strong inflammatory profile compared to peripheral blood neutrophils (PBNs). These tumor-associated neutrophils (TANs) seem to adapt to the unique environment inside the brain. Through a series of experiments and analyses, they proved that the brain TME influences TANs in releasing specific molecules. One of the most striking effects of the brain TME on TANs is their extended lifespan compared to PBNs [25]. 

### 2.2. Non-Immune Component Cells

Blood–Brain Barrier (BBB). The BBB maintains CNS homeostasis through a tightly regulated neurovascular unit comprising endothelial cells, pericytes, and astrocytes. Functioning as a selective barrier, the BBB limits the passive diffusion of molecules between the brain and systemic circulation. This protective mechanism allows only essential substances to enter the brain, while immune cells can access the brain tissue through specific passage points located downstream of capillaries (post-capillary venues). Yet, conversely to what was thought previously, the BBB does not act as a “hermetic seal”, permitting T-cell entry and immunosurveillance [26]. In GBM, the BBB is disrupted, leading to increased and heterogeneous permeability, resulting in a blood–tumor barrier (BTB) that facilitates immune cell efflux into the TME [27].

Astrocytes. Astrocytes play a crucial role in maintaining healthy brain function by providing structural support and preserving the BBB [28]. In a healthy CNS, they also contribute to tissue repair following injury by secreting growth factors and cytokines [29]. However, in the TME, this repair process called astrogliosis or reactive gliosis can support tumor growth and promote resistance to glioma therapies [30]. Astrocytes exhibit different phenotypes, depending on the surrounding microenvironment. The “A2” astrocyte, which is associated with an anti-inflammatory environment, promotes repair and homeostasis, while the “A1” astrocyte, responding to pro-inflammatory signals, is involved in immune responses, like antigen presentation and complement activation. In the GBM TME, astrocytes interact with microglia, activating pathways, such as JAK/STAT and PD-L1, which in turn elevate anti-inflammatory cytokines, like IL-10, TGF-β, and STAT3. This leads to an immunosuppressive cold tumor environment that hinders effective immune responses [31].

Oligodendrocyte precursor. Adult oligodendrocyte precursor cells (OPCs) are the most proliferative cells in the CNS, making up about 10% of human brain cells [32]. While their primary role is to generate myelinating oligodendrocytes [33], OPCs also have various myelination-independent functions in both healthy and diseased nervous systems [34]. In particular, OPCs are the main cell type involved in glioma development [35]. A recent work conducted on GBM murine models showed that OPCs significantly contribute to glioma formation [36]. In gliomas with isocitrate dehydrogenase (IDH) variants, genes regulating OPC specification and maintenance are upregulated, while genes involved in myelination are downregulated, indicating that these tumors resemble early-stage OPC development [35]. In this context, the fact that OPCs respond to neural activity [37] is relevant because it has been shown that glutamatergic synaptic inputs to glioma cells drive both glioma progression and migration of GBM cells [38,39,40].

Vasculature. GBM is characterized by aberrant vasculature, edema, hypoxia, and necrosis. Hypoxia activates angiogenesis, enhances tumor survival, suppresses antitumor immunity, and hinders therapeutic response. Oxygen levels in the brain typically range from 12.5% to 2.5%, but a hallmark of the GBM TME is chronic hypoxia, with oxygen levels dropping as low as 0.1% [41]. This hypoxic condition, driven by rapid tumor cell proliferation and inadequate blood vessel formation, profoundly impacts various aspects of cancer biology, including chemotherapy resistance, angiogenesis, increased cell proliferation, and tissue invasion. GBM cells thrive in this oxygen-deprived environment through the expression of hypoxia-inducible factors (HIFs), particularly HIF-1α and HIF-2α. Elevated levels of these factors are associated with poorer prognosis and higher mortality risk in brain tumor patients [42]. Experimental studies further confirm that increased HIF-1α expression promotes tumor growth, while HIF-1α suppression reduces it. Additionally, GBM cells undergo metabolic reprogramming, allowing them to generate energy and adapt to the harsh TME, facilitating their invasion of healthy brain tissue and enabling tumor spread.

Glioma Stem Cells (GSCs). Glioma Stem Cells (GSCs) are recognized as the primary drivers of GBM growth and recurrence. Their inherent capacity for self-renewal, proliferation, differentiation, and the initiation and maintenance of tumors contributes significantly to the aggressive nature of this malignancy. This self-renewal capacity is further underscored by their resistance to standard-of-care chemotherapy, particularly TMZ, which significantly impacts treatment outcomes. Notably, GSCs not only exhibit intrinsic resistance to TMZ due to the high expression of the ABCB4 efflux pump, particularly evident in CD133-positive GSCs, but also contribute to the development of a broader tumor resistance. This occurs through the transfer of ABCB4 protein to differentiated glioma cells via exosomes, effectively fostering a microenvironment resistant to TMZ therapy within the tumor [43,44,45].

Neurons. The neurons are excitable and specific cells of nervous tissue. Growing evidence highlights a complex, bidirectional interaction between the nervous system and cancer, including GBM, where nervous system structures can influence tumor growth, while tumors in turn can affect neural function and structure [46]. Recent studies reveal that neurons and glioma cells are engaged in synaptic communication, with neurotransmitter release and voltage-gated mechanisms enabling tumor cells to exploit neuronal signals for growth [47]. Literature data reported that neurons support glioma progression by upregulating neuroligin-3 (NLGN3), which induces a phosphoinositide-3-kinase (PI3K) signaling-mediated proliferative activity in glioma cells [39]. NLGN3 expression in GBM is inversely correlated with patient survival, and neuron–glioma interactions promote glioma proliferation through synaptic and non-synaptic mechanisms. Furthermore, it was demonstrated that altering neuronal activity can change the number of dividing tumor cells. This growth appears to be initially driven by substances released by neurons, such as brain-derived neurotrophic factor (BDNF) and NLGN3 [48].

Extracellular matrix. The brain extracellular matrix (ECM) contains distinctive components that are not found in other tissues. It consists of relatively small amounts of fibrous proteins, such as collagen, fibronectin, and laminins, but a higher concentration of glycosaminoglycans. These glycosaminoglycans may be protein bound (including chondroitin sulfate, dermatan sulfate, heparan sulfate, and keratan sulfate) or exist as free hyaluronan. Additionally, the ECM contains proteoglycans, known as lecticans, such as versican, aggrecan, neurocan, brevican, and decorin, along with glycoproteins, like tenascin-C [49]. The brain tumor ECM has a complex composition that differs significantly from that of healthy brain tissue. An increasing amount of research has highlighted the pivotal roles the brain tumor ECM plays in the development of GBM, as well as proposed various innovative strategies to target ECM components and pathways associated with GBM to hinder tumor growth and overcome therapy resistance. However, many of these approaches remain in the preclinical development phase [50]. Both in vitro functional studies and genetic research in animal models have shown that the ECM is integral to nearly every aspect of nervous system development and function. Moreover, it plays a pivotal role in all stages of GBM progression. In GBMs, the ECM undergoes considerable remodeling compared to that of healthy brain tissue. Emerging evidence highlights that the three-dimensional ECM architecture and its mechanical properties influence cellular behavior within the TME, both at the tumor core and in the surrounding areas. ECM components can act as either attractants or repellents to glioma cells, microglia, monocytes, macrophages, and stem cells, all of which are key constituents of the TME [51]. Key ECM components like hyaluronic acid, tenascin-C, laminin, and collagen influence how invasive tumor cells become. GBM tumor cells, in particular, can remodel and break down the ECM. They achieve this by releasing enzymes called matrix metalloproteinases (MMPs) into the surrounding space. This modification of the ECM by GBM cells further promotes their infiltration into healthy brain tissue [52]. In addition, although collagen is normally found at low levels in healthy brain tissue, GBM tumors exhibit increased collagen expression. This increase in collagen plays a significant role in how GBM cells move and invade surrounding tissues [13].

## 3. Purinergic Landscape in GBM

Purine molecules, including adenosine 5′-triphosphate (ATP) and adenosine, are increasingly recognized for their vital roles in cellular and extracellular functions [53]. Beyond regulating energy metabolism and other processes within cells, purines bind to extracellular purinergic receptors, activating signaling pathways that influence diverse physiological and pathological processes such as development, aging, regeneration, and inflammation [54].

The “purinergic hypothesis”, introduced in the 1970s [55,56], spurred extensive research into purinergic signaling under various conditions [57]. This research has highlighted its relevance in tumor biology, paving the way for therapeutic interventions targeting purinergic pathways. The translation of these findings into tailored drugs is now a growing focus in clinical applications.

Purinergic receptors, identified in 1972, are classified into two categories: P1 receptors (P1Rs) and P2 receptors (P2Rs).

- P1Rs, a class of G protein-coupled receptors (GPCRs), are primarily activated by adenosine. Subtypes (A1, A2A, A2B, A3) exhibit distinct tissue distributions and activation thresholds, with A2BR functioning at lower adenosine concentrations than A1AR and A2AR [58].
- P2Rs, activated by ATP or other nucleotides, are further divided into P2X receptors (P2XRs) and P2Y receptors (P2YRs).
  ○ P2YRs, also GPCRs, regulate intracellular calcium and cyclic adenosine monophosphate (cAMP) levels, responding to agonists beyond ATP.
  ○ P2XRs are ATP-dependent, ligand-gated, cation-selective channels, with P2X7R uniquely forming a macropore that internalizes large molecules up to 900 Da.

These receptors, expressed across neuronal and non-neuronal cells, regulate critical processes in health and disease [59,60]. Understanding the interplay between purinergic signaling, cancer progression, and immune pathways in the TME is essential for developing novel therapeutic strategies.

The GBM TME is known for its accumulation of adenosine [61]. Adenosine, a purine nucleoside, plays various roles in healthy cells, including suppressing immune cell activity, regulating nerve cell function, and reducing pain perception. In normal conditions, adenosine concentration ranges from 30 to 200 nM. However, in pathological conditions, like GBM, its concentration can surge to much higher levels, reaching 1 to 10 µM. This molecule is produced through a step-by-step breakdown of ATP, the cell’s main energy source. Enzymes like CD39 and CD73 play a key role in this process. This conversion creates an environment abundant with growth-promoting and immunomodulatory factors [62]. An in silico analysis carried out by the group of Braganhol revealed also that both CD73 and CD39 are overexpressed in GBM tumor tissue compared to the normal brain or non-tumor brain tissue, illustrating that the expression of the purinergic system in GBM is different from the one expressed in non-tumor tissue. Furthermore, the degree of the expression of CD73 and CD39 is related to a shorter median survival rate in patients with GBM [63]. 

Regarding CD73, several studies showed its role in regulating NK infiltration in the TME. Specifically, extracellular adenosine induces the inhibition of proliferation, maturation, and cytotoxic function in tumor-infiltrating NK cells [64] in GBM as well [65]. 

In recent years, experimental evidence has shown that ATP can be released into the TME through various mechanisms, where it regulates signaling pathways via P2 [66] purinergic receptors and triggers a cascade of reactions. In general, eATP levels, which are typically maintained in a homeostatic balance under physiological conditions, are elevated in pathological states, such as tumors. The accumulation of eATP in the TME arises from multiple sources and mechanisms. First, passive release occurs due to cellular damage from inflammation, hypoxia, chemotherapy, and tissue destruction from tumor invasion. Second, active secretion of ATP from living or apoptotic cells via vesicular exocytosis, transporters, or membrane-bound pathways further contributes to elevated eATP concentrations. In the TME, eATP levels can rise to several hundred micromoles, transmitting active signals that influence tumor cell metabolism and immunity. The specific role of eATP in the TME is determined by both its concentration and the associated signaling pathways.

Interestingly, hypoxic regions, inflammation, and pharmacological treatments can lead to a constant release of eATP, explaining the different expression of ATP and ADP from normal to cancer tissue [42,62,67,68].

These findings highlight the role of purinergic mediators in the intricate interplay between tumor cells, microenvironment, and immunity.

## 4. P2X7 Receptor in the GBM TME

**Figure 2:**
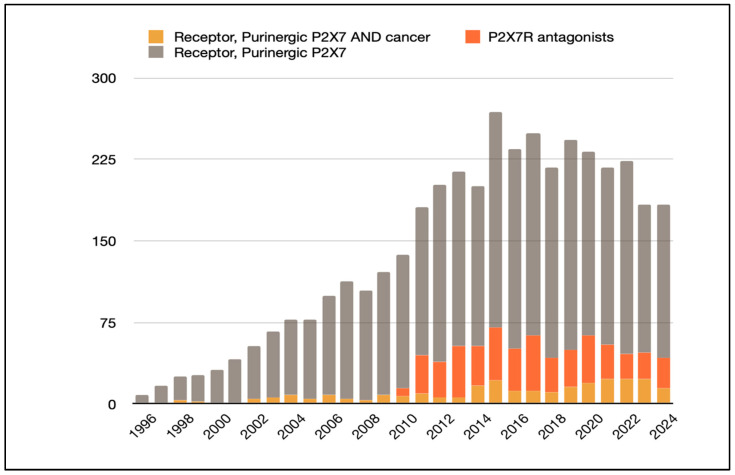
Purinergic P2X7 receptor in the literature. Search for articles appearing in PUBMED over the past 28 years (1996–2024) using the Mesh terms “Receptor, purinergic P2X7 AND Neoplasms”, “Antagonists, P2X7 purinoreceptor”, and “Receptor, purinergic P2X7”.

Among purinergic receptor subtypes, P2X7R stands out due to its unique characteristics, including low ATP affinity, dual functionality upon activation, and its ability to mediate ion channel activity and form non-selective pores. These features make P2X7R a promising target for antitumor therapy, particularly in patients who respond poorly to immunotherapy, and it has been gaining increasing importance in the scientific community since the 2000s (Figure 2).

P2X7R plays a critical role in tumor progression, influencing cell proliferation and apoptosis, thus positioning it as a viable therapeutic target. Current research focuses on harnessing the unique properties of P2X7R in the TME to develop safe and effective immunotherapies, as evidenced by the growing body of literature on novel antagonist development (Figure 2).

The role of P2X7R in the TME is influenced by several factors, including the concentration and timing of agonists, cell type, and host–tumor interactions. In particular, P2X7R plays a dual role depending on the concentration and duration of eATP stimulation. Low concentrations or transient stimulations of eATP allow P2X7R to open channels for small cations, promoting tumor cell proliferation and enhancing immune responses. Conversely, high concentrations or sustained eATP exposure result in non-selective macropore opening, enabling the entry of larger ions and inflammatory cytokines. This can lead to irreversible damage, ultimately causing tumor cell lysis and death. Developing immune-targeting drugs requires careful consideration of the basal expression levels of P2X7R. Additionally, the heterogeneity of immune cell distribution and context within the TME across different tumor types significantly impacts the efficacy of antitumor immunotherapies, necessitating tailored approaches to treatment.

Interestingly, in the GBM TME, P2X7R expression differs between the tumor core and the peripheral tissue. Particularly, it was demonstrated that the receptor is present in both GBM tumors and microglia cells, and its expression is higher in microglia found within the tumor compared to those in healthy tissue surrounding the tumor [69].

As already mentioned, the ATP levels in the TME are higher than in healthy compartments, and in gliomas, ATP is degraded slowly, increasing its level in tumor regions [70,71]. Additionally, several types of immune cells express P2X7R, which influences them through several intracellular signaling pathways. Among others, loss of P2X7R causes a reduction in CD8+ T-cell infiltration in a murine cancer model, and a high expression of the receptor on monocytes and macrophages is involved in immune suppression [70]. Otherwise, the activation of P2X7R by an elevated concentration of ATP in glioma induces the production of chemokine, which could mobilize microglia and macrophages into tumors [71]. Furthermore, a recent study [12] revealed that TAMs enhance mitochondrial activity and eATP production in the GBM microenvironment, promoting glioma growth and invasion via P2X7 receptor activation. Inhibiting P2X7R was shown to reduce tumor growth, suggesting the eATP–P2X7R pathway is a promising therapeutic target for GBM.

Drill et al. [72] identified a novel link between P2X7R and GM-CSF, and their findings support P2X7R inhibition as a potential therapeutic approach for GBM. In their in vitro model, they demonstrated that the P2X7R antagonist AZ10606120 significantly reduced GM-CSF levels and GBM cell proliferation, exhibiting a superior tumor-killing effect compared to TMZ, the current first-line treatment for GBM.

Similarly, Kan et al. [73] confirmed the functional expression of P2X7R in GSCs and showed that inhibiting P2X7R with AZ10606120 significantly decreased GSC numbers and induced cytotoxicity, surpassing the efficacy of TMZ chemotherapy. These findings collectively suggest that targeting P2X7R inhibition may offer a promising therapeutic strategy to overcome GSC resistance in GBM.

Moreover, it was reported that GSCs released in TME extracellular vesicles (EVs) enriched with proteins influenced tumor aggressiveness, with their composition significantly altered by P2X7R stimulation [74]. In particular, P2X7R activation induces proteins associated with cytoskeleton reorganization, cell motility, energy supply, and transcriptional regulation, many of which are linked to GBM progression [74]. These findings suggest P2X7R as a potential therapeutic target for modulating the TME and secretome in GBM.

In light of this evidence, targeting P2X7R could be a key for GBM TME remodeling and cancer progression. For this reason, several antagonists have been studied.

D’Alimonte and colleagues [75] showed that purine receptor ligands, particularly ATP and BzATP, enhance the antitumor efficacy of TMZ against GSCs by inducing growth arrest, apoptosis, and necrosis while suppressing secondary neurosphere formation. These findings suggest a novel combination approach targeting A3R, P2Y1R, and P2X7R receptors for improved and long-lasting GBM therapy. Gehring et al. [76] reported that P2X7R is crucial for glioma radiosensitivity, as its activation enhances radiotherapy-induced cytotoxicity, while P2X7R silencing impairs this effect in vitro and in vivo. High P2X7R expression correlates with improved radiotherapy response and serves as a positive prognostic marker for glioma patient survival.

The work of Kwak et al. [77], starting from the screening quinolinone derivatives, identified compound 11a as a P2X7 receptor antagonist, with the structure–activity relationship (SAR) analysis revealing key substituents for optimal activity. Further optimization of the core structure led to quinoline-based antagonists 16c and 17k, which exhibited potent inhibition of P2X7R with IC_50_ values of 4 and 3 nM, respectively. These antagonists also inhibited IL-1β release and reduced GBM cell sphere size, highlighting their potential as therapeutic agents.

A more recent work delves deeper into the role of P2X7R in radioresistance using patient-derived GBM models. Zanoni et al. [78] showed that radiation induces a shift in P2X7R isoform expression in GBM stem cells, with P2X7A downregulated and P2X7B upregulated, promoting growth and survival post-irradiation. Blocking P2X7B during recovery enhances irradiation-dependent cytotoxicity, highlighting its role in radiotherapy resistance. Furthermore, Sun et al. [79] highlighted the upregulation of CD39 expression induced by ionizing radiation via the STAT1-IRF1-CD39 axis, creating an immunosuppressive TME and promoting radioresistance. CD39 blockade increases extracellular ATP, activating dendritic cells through P2X7Rs to enhance radiation-induced immunogenic cell death (ICD). Combining CD39 inhibition with ICD-based vaccines boosts CAR-T cell expansion, persistence, and antitumor efficacy in glioma mouse models, overcoming challenges of antigen loss in conventional CAR-T therapies. Ziberi et al. [80] demonstrated in GSC models that high P2X7R expression, activated by BzATP, drives epithelial-to-mesenchymal transition (EMT), enhancing GSC migration, invasiveness, and survival via the SMAD2 pathway. P2X7R activation also upregulates its A and B splice variants, likely increasing receptor sensitivity and metabolic support for GSCs. These findings position P2X7R as a crucial factor in GBM recurrence and invasiveness, proposing its inhibition as a potential therapeutic approach. Furthermore, GBM exhibits the upregulation of P2X7R and GM-CSF, with P2X7R activity shown to drive GM-CSF expression in U251 GBM cells. P2X7R antagonism using AZ10606120 reduces GM-CSF levels and significantly decreases GBM cell proliferation. These findings reveal a novel P2X7R-GM-CSF link and support P2X7R inhibition as a potential therapeutic strategy for GBM.

GBM cells generally have a low tumor mutation burden (TMB) [81], resulting in limited neoantigen presentation for effective T-cell recognition. Since neoantigen presentation relies on the presence of mutations that generate neoepitopes, the scarcity of such mutations restricts immune activation [81]. While some mutations can be immunogenic and presented by antigen-presenting cells (APCs), most do not become MHC-presented neoepitopes recognized by T-cells [82]. Additionally, tumor subclones lacking highly antigenic peptides may escape immune detection [82]. Consequently, the low TMB reduces the pool of immunogenic neoantigens available after immunotherapy, which aligns with the poor clinical efficacy of checkpoint inhibitors in GBM trials [83].

In contrast, the immune microenvironment of GBM is heavily infiltrated by TAMs comprising almost 40% of the tumor mass, with functional and metabolic heterogeneity, influencing glioma progression either positively or negatively [84]. A recent study by Wu et al. (2024) reported increased ATP synthase expression and oxidative phosphorylation activity in TAMs within the GBM tumor core compared to the periphery [12]. Both in vitro and in vivo models demonstrated heightened mitochondrial activity in TAMs, characterized by increased mitochondrial fission, glucose uptake, membrane potential, and eATP production in response to GBM cells. Tumor-secreted factors, particularly GM-CSF, were identified as drivers of elevated eATP levels. This, in turn, promoted glioma growth and invasion via activation of P2X7R on glioma cells. Notably, inhibiting the eATP–P2X7R signaling pathway reduced tumor cell viability in vitro, decreased tumor size, and extended survival in glioma-bearing mice. These findings highlight TAM-derived eATP as a potential therapeutic target in GBM [12].

Overall, purinergic receptors, particularly P2X7R, are crucial in the modulation of inflammation, cell death, and immune suppression in GBM. Several studies have confirmed that the activation of P2X receptors can initiate various signaling pathways and induce an inflammatory response [85]. Nonetheless, understanding the intracellular signaling pathways activated by this receptor is still in the early stages. As previously mentioned, the abundant eATP in the GBM TME, released by neurons and stressed cells, activates P2X7R. This activation increases ROS production and induces mitochondrial depolarization, contributing to the maintenance of the cancer phenotype [86]. Furthermore, the activation of the receptor is associated with numerous cell-specific signal transduction pathways, including PKC/MEK/ERK/FOS/JUN, PI3K/AKT/mTOR, MyD88/NF-κB, MMP-2/9, and calcineurin/NFATc1 [87]. These pathways are frequently involved in the release of inflammatory mediators such as caspase-1, IL-1β, IL-6, TNF-α and NLRP3/ASC. By activating these signaling pathways, P2X7R plays an important role in promoting an inflammatory milieu, metastasis, angiogenesis, and invasion [88]. Additionally, it stimulates MDSCs to release immunosuppressive factors and induces macrophage polarization toward the M2 phenotype. Furthermore, the network of ectoenzymes regulating extracellular nucleotide metabolism remains largely unexplored in gliomas. Recent findings indicate that the P2X7R/CD39/CD73 axis plays a crucial role in modulating the immune landscape of the TME. Notably, glioma cells, regardless of grade, overexpress CD73 while exhibiting low CD39 expression [89]. Concurrently, targeting CD39 can enhance P2X7R activation, boosting antitumor responses through mechanisms beyond simply inhibiting adenosine formation [87]. 

## 5. Conclusions

GBM shows significant treatment challenges, including its aggressive infiltration into healthy tissue and the BBB, which limits therapeutic delivery and immune response. Additionally, the CNS immune system, once thought to be isolated, is now recognized as functional but uniquely structured, influencing immune responses in a distinct manner. The GBM TME is characterized by profound immunosuppression, which not only suppresses endogenous antitumor immunity but also limits the effectiveness of immunotherapies, contributing to its unique and resistant nature.

**Figure 3:**
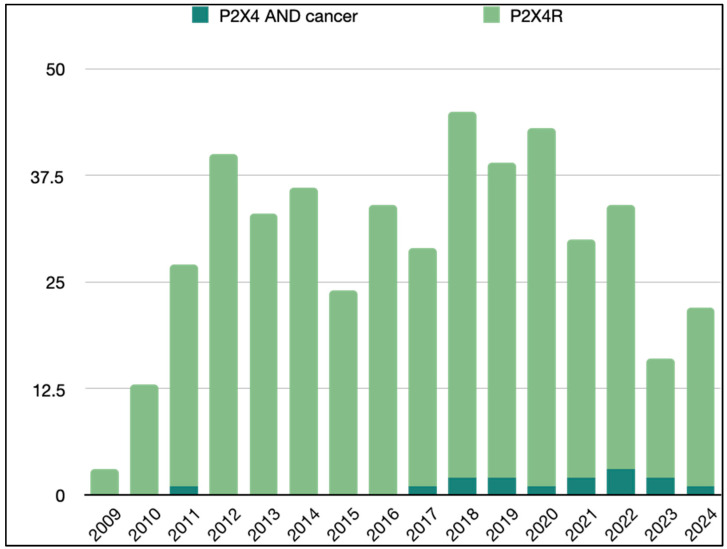
Purinergic P2X4 receptor in the literature. Search for articles appearing in PUBMED over the past 15 years (2009–2024) using the Mesh terms “Receptor, purinergic P2X4 AND Neoplasms” and “Receptor, purinergic P2X4”.

In this context, purinergic signaling, particularly through P2X7R, has been emerging as a key player in GBM progression. This review deepened the understanding of purinergic signaling in the GBM TME and explored targeting the P2X7R pathway as a potential therapeutic strategy to overcome GBM immune evasion and resistance to treatment. However, other key components of the purinergic signaling network may play a critical role in the uncontrolled proliferation and inevitable progression of GBM. Among these, the P2X4 receptor (P2X4R), able to bind to eATP, stands out as particularly promising, with increasing evidence linking its involvement to the pathogenesis of various tumor types, including GBM (Figure 3). P2X4R is predominantly expressed in microglia within the central nervous system, and its activation has been linked to the release of pro-inflammatory cytokines and the development of neuropathic pain. Furthermore, it can modulate the activity of microglia, contribute to the progression of neuroinflammation, and play a crucial role in maintaining nervous system functions [85]. While its direct role in GBM is less well defined compared to P2X7R, P2X4R contributes to the inflammatory environment that can influence tumor progression [90]. Indeed, the expression of both P2X4R and P2X7R induces P2X7R-mediated IL-1β release and inflammation by facilitating calcium influx, thereby amplifying the inflammatory response. This suggests that P2X4R expression is essential for P2X7R-dependent IL-1β release [85].

Notably, Guo et al. [91] demonstrated that P2X4R was highly expressed in C6 glioma and highly expressed in all areas of tumor growth. In addition, Huo et al. reported that silencing P2X4R in GBM cell lines (T98 and U87) impaired cell viability and proliferation while promoting apoptosis through increased caspase-3 activity. Further investigation revealed that P2X4R suppression inhibited the BDNF/TrkB/ATF4 signaling pathway, with the effects on GBM cell growth and apoptosis being reversible by ATF4 overexpression, highlighting P2X4R as a critical regulator of GBM progression [92]. Moreover, P2X7R activation has been shown to engage multiple inflammatory intracellular signaling pathways, including the NLRP3 inflammasome, the PI3K/Akt pathway, and NF-κB activation, leading to the release of IL-1β in GBM. In this context, the co-expression of P2X4R and P2X7R facilitates calcium influx, inducing P2X7R-mediated IL-1β release and enhancing the inflammatory response [85]. Therefore, P2X4R potentially interacts with P2X7R to further amplify these inflammatory responses [72], highlighting its essential role in P2X7R-dependent IL-1β release. Thus, both P2X7R and P2X4R are involved in glioma cell proliferation and survival, yet they trigger different signaling pathways. While P2X7R primarily activates pro-survival pathways, such as p38 MAPK and Akt [86], P2X4R engages the BDNF/TrkB pathway [92].

Given these promising findings, we anticipate the development and testing of brain-permeant anti-P2X4R molecules for GBM in the near future, potentially offering a novel therapeutic approach for this aggressive tumor.

Currently, only a few P2X7R antagonists are under clinical trials and only for neuroinflammatory diseases. Janssen Pharmaceuticals has recently developed a selective P2X7R antagonist that has undergone clinical trials to evaluate its potential in treating major depressive disorder [93]. Two other clinical trials on rheumatoid arthritis [94] and inflammatory pain [95] are investigating the role of the P2X7R antagonist. Regarding the P2X4R antagonist, NC-2600 entered a Phase 1 clinical trial for chronic neuropathic pain in Japan in June 2016 [96] (Table 1).

Although these drugs are not specific against GBM, they could be interesting in relieving neuroinflammation and cognitive impairment caused by chemo- and radiotherapy [70,97]. However, these compounds could be the baseline to study similar molecules useful for GBM treatment.

## Figures and Tables

**Table 1:** Clinical trials of P2X receptor antagonists.

Antagonist	Target Receptor	Disease	Mechanism of Action
JNJ-54175446	P2X7R antagonist	Major depressive disorder	Selective P2X7R antagonist. It prevents ATP binding, thereby inhibiting downstream inflammatory signaling. Furthermore, it reduces IL-1β release and modulates neuroinflammation [ClinicaTrials.gov NCT04116606].
GSK1482160	P2X7R antagonist	Inflammatory pain	Selective and orally available P2X7R allosteric modulator. It prevents sustained ATP activation, blocking the formation of large pores that would lead to membrane disruption and cytokine release. Reduces IL-1β and IL-18 release and has neuroprotective and anti-inflammatory effects [ClinicalTrials.gov NCT00849134].
CE-224,535	P2X7R antagonist	Rheumatoid arthritis	Selective P2X7 receptor antagonist. It prevents ATP binding and plays a role in macrophage and monocyte activation, dampening immune overactivation [ClinicalTrials.gov NCT00628095].
NC-2600	P2X4R antagonist	Neuropathic pain	An orally administered and selective antagonist of the P2X4R. The activation of P2X4R by eATP leads to calcium influx and the subsequent release of pro-inflammatory cytokines, contributing to neuropathic pain and inflammation. NC-2600 dampens microglial activation, thereby alleviating pain hypersensitivity [Jacobson, K.A., et al. Treatment of chronic neuropathic pain: Purine receptor modulation. Pain 2020, 161, 1425–1441].

## Abbreviations

The following abbreviations are used in this manuscript:

GBM	Glioblastoma
TME	Tumor Microenvironment
CNS	Central Nervous System
TMZ	Temozolomide
BBB	Blood–Brain Barrier
TAMs	Tumor-Associated Macrophages and Microglia
eATP	Extracellular ATP
P2X7R	P2X Purinoceptor 7
MCP	Microenvironment Cell Population
GAMs	Glioma-Associated Microglia and Macrophages
TILs	Tumor-Infiltrating Lymphocytes
NK	Natural Killer
MDSCs	Myeloid-Derived Suppressor Cells
PBNs	Peripheral Blood Neutrophils
TANs	Tumor-Associated Neutrophils
BTB	Blood–Tumor Barrier
OPCs	Oligodendrocyte Precursor Cells
IDH	Isocitrate Dehydrogenase
HIFs	Hypoxia-Inducible Factors
GSCs	Glioma Stem Cells
NLGN3	Neuroligin-3
PI3K	Phosphoinositide-3-kinase
BDNF	Brain-Derived Neurotrophic Factor
ECM	Extracellular Matrix
MMPs	Metalloproteinases
ATP	Adenosine 5’-Triphosphate
P1Rs	P1 Receptors
P2Rs	P2 Receptors
GPCRs	G Protein-Coupled Receptors
P2XRs	P2X Receptors
P2Yrs	P2Y Receptors
cAMP	Cyclic Adenosine Monophosphate
GM-CSF	Granulocyte Macrophage Colony-Stimulating Factor
EVs	Extracellular Vesicles
SAR	Structure–Activity Relationship
ICD	Immunogenic Cell Death
EMT	Epithelial-To-Mesenchymal Transition
TMB	Tumor Mutation Burden
APC	Antigen-Presenting Cell
