# Correction: Bedeschi et al. Glioblastoma Tumor Microenvironment and Purinergic Signaling: Implications for Novel Therapies. *Pharmaceuticals* 2025, *18*, 385

**DOI:** 10.3390/ph18111742

**Published:** 2025-11-17

**Authors:** Martina Bedeschi, Elena Cavassi, Antonino Romeo, Anna Tesei

**Affiliations:** 1Biosciences Laboratory, IRCCS Istituto Romagnolo per lo Studio dei Tumori (IRST) “Dino Amadori”, 47014 Meldola, Italy; martina.bedeschi@irst.emr.it (M.B.); elena.cavassi@irst.emr.it (E.C.); 2Radiation Oncology Unit, IRCCS Istituto Romagnolo per lo Studio dei Tumori (IRST) “Dino Amadori”, 47014 Meldola, Italy; antonino.romeo@irst.emr.it

## References

In the original publication [1], the reference 22 was retracted and it has been replaced the new reference as below:22.Miller, T.E.; El Farran, C.A.; Couturier, C.P.; Chen, Z.; D’Antonio, J.P.; Verga, J.; Villanueva, M.A.; Gonzalez Castro, L.N.; Tong, Y.E.; Saadi, T.A.; et al. Programs, origins and immunomodulatory functions of myeloid cells in glioma. *Nature* **2025**, *640*, 1072–1082. https://doi.org/10.1038/s41586-025-08633-8.

This correction was approved by the Academic Editor, and the authors state that their scientific conclusions remain unaffected. The original publication has also been updated.
